# Impaired SARS-CoV-2-Specific CD8+ T Cells After Infection or Vaccination but Robust Hybrid T Cell Immunity in Patients with Multiple Myeloma

**DOI:** 10.3390/vaccines12111249

**Published:** 2024-11-01

**Authors:** Khalid Shoumariyeh, Benedikt Csernalabics, Elahe Salimi Alizei, Matthias Reinscheid, Sebastian Giese, Kevin Ciminski, Georg Kochs, Martin Schwemmle, Julia Lang-Meli, Michelle Maas, Natascha Roehlen, Vivien Karl, Anne Graeser, Oezlem Sogukpinar, Ivana von Metzler, Denise Grathwohl, Leo Rasche, Holger Hebart, Miriam Kull, Florian Emmerich, Cornelius Florian Waller, Justus Duyster, Monika Engelhardt, Tanja Nicole Hartmann, Bertram Bengsch, Tobias Boettler, Christoph Neumann-Haefelin, Maike Hofmann, Robert Thimme, Hendrik Luxenburger

**Affiliations:** 1 Department of Medicine I, Medical Center—University of Freiburg, Faculty of Medicine, University of Freiburg, 79098 Freiburg, Germany; 2 German Cancer Consortium (DKTK), Partner Site Freiburg, a Partnership Between DKFZ and University Medical Center Freiburg, 79098 Freiburg, Germany; 3 Department of Medicine II (Gastroenterology, Hepatology, Endocrinology and Infectious Diseases), Freiburg University Medical Center, Faculty of Medicine, University of Freiburg, 79098 Freiburg, Germany; 4 Faculty of Chemistry and Pharmacy, University of Freiburg, 79098 Freiburg, Germany; 5 Faculty of Biology, University of Freiburg, 79098 Freiburg, Germany; 6 Institute of Virology, Freiburg University Medical Center, Faculty of Medicine, University of Freiburg, 79098 Freiburg, Germany; 7 Department of Medicine II—Hematology and Oncology, Goethe-University Frankfurt, University Hospital, 60629 Frankfurt am Main, Germany; 8 Frankfurt Cancer Institute (FCI), 60596 Frankfurt am Main, Germany; 9 German Cancer Consortium (DKTK), Partner Site Frankfurt/Mainz, a Partnership Between DKFZ and University Hospital Frankfurt, 60596 Frankfurt am Main, Germany; 10 Department of Internal Medicine II, University of Würzburg, 97070 Würzburg, Germany; 11 Clinics Ostalb, Stauferklinikum, 73557 Mutlangen, Germany; 12 Department of Internal Medicine III, Ulm University Hospital, 89081 Ulm, Germany; 13 Institute for Transfusion Medicine and Gene Therapy, Freiburg University Medical Center, Faculty of Medicine, University of Freiburg, 79098 Freiburg, Germany; 14 Signalling Research Centres BIOSS and CIBSS, University of Freiburg, 79098 Freiburg, Germany

**Keywords:** multiple myeloma, immunosuppression, COVID-19, SARS-CoV-2, mRNA vaccination, infection, T cells, adaptive immune response

## Abstract

Background: Multiple myeloma (MM) patients are at high risk of severe infections including COVID-19 due to an immune dysregulation affecting both innate and adaptive immune responses. However, our understanding of the immune responses to infection and vaccination in MM patients is limited. To gain more detailed insights into infection- and vaccine-elicited T cell immunity in MM, we studied the CD8+ T cell response on the single-epitope level in SARS-CoV-2 convalescent and mRNA-vaccinated MM patients. Methods: We compared peptide/MHC class I tetramer-enriched SARS-CoV-2-specific CD8+ T cells and antibody responses in MM patients (convalescent: *n* = 16, fully vaccinated: *n* = 5, vaccinated convalescent: *n* = 5) and healthy controls (HCs) (convalescent: *n* = 58, fully vaccinated: *n* = 7) either after infection with SARS-CoV-2 alone, complete mRNA vaccination or SARS-CoV-2 infection and single-shot mRNA vaccination (hybrid immunity). Results: MM patients have lower frequencies and a lower proportion of fully functional virus-specific CD8+ T cells compared to HCs, after both SARS-CoV-2 infection and vaccination. CD8+ T cell memory subset distribution in MM patients is skewed towards reduced frequencies of central memory (T_CM_) T cells and higher frequencies of effector memory 1 (T_EM1_) T cells. In contrast, the humoral immune response was comparable in both cohorts after viral clearance. Notably, CD8+ T cell frequencies as well as the humoral immune response were improved by a single dose of mRNA vaccine in convalescent MM patients. Conclusions: MM patients have relative immunological deficiencies in SARS-CoV-2 immunity but benefit from hybrid immunity. These findings underline the relevance of vaccinations in this vulnerable patient group.

## 1. Introduction

Patients with multiple myeloma (MM) have an increased risk of bacterial and viral infections compared to the general population [1,2], caused by plasma cells infiltrating the bone marrow and altering both innate and adaptive immunity [3]. With regard to viral infections of the respiratory tract, such as influenza [2,4] or SARS-CoV-2 [5,6,7,8], MM is associated with a more severe course of infection and a reduced efficacy of vaccines compared to the general population.

However, only limited data are available assessing the immune response induced by infections and vaccinations in MM patients. For example, after SARS-CoV-2 vaccination, most studies in MM patients have focused on humoral immunity, showing reduced titers of spike-specific immunoglobulin G (IgG) and neutralizing antibodies in MM patients [9,10,11], while cellular immunity is hardly characterized compared to immunocompetent individuals. In immunocompetent individuals, SARS-CoV-2 infection and vaccination have been shown to induce all components of the adaptive immune system including neutralizing antibodies and T cells [12,13,14,15,16,17,18,19]. Hence, careful analysis of the cellular immunity including CD8+ T cells in MM patients may have important implications for future vaccination strategies in the vulnerable group of patients with hematologic malignancies. Therefore, the aim of this study was to investigate SARS-CoV-2-related immunity, in order to assess the adaptive immune response elicited by infection and vaccination in MM patients compared to healthy controls (HCs). By this, we here provide an in-depth profiling of the CD8+ T cell response at the single-epitope level in MM patients and highlight the benefit of hybrid immunity for this vulnerable patient group.

## 2. Materials and Methods

### 2.1. Study Cohort

A total of 16 convalescent MM patients (including 5 with a single mRNA vaccination during follow-up) and 51 convalescent HCs as well as 5 MM patients and 7 HCs who received a complete vaccination with three mRNA vaccine doses were recruited at the University Medical Center Freiburg (Germany), University Hospital Frankfurt (Germany) and the University Hospital of Würzburg (Germany). Severity of infection was characterized according to the WHO classification. SARS-CoV-2 infection was confirmed by positive PCR testing from oropharyngeal swab and/or SARS-CoV-2 spike IgG-positive antibody testing. HLA typing was performed by next-generation sequencing. Characteristics of the cohorts are summarized in Table 1, Table 2 and Table S1.

### 2.2. PBMC Isolation

Blood was taken from study participants and supplemented with EDTA to prevent coagulation. PBMCs were isolated by density gradient centrifugation using Pancoll separation medium (PAN Biotech GmbH, Aidenbach, Germany). Until further processing, PBMCs were stored at −80 °C and thawed upon demand using RPMI 1640 supplemented with 10% fetal calf serum, 1% penicillin/streptomycin, 1.5% HEPES buffer 1 M (all Thermo Scientific, Waltham, MA, USA) and 50 U mL^−1^ benzonase (Sigma, St. Louis, MI, USA).

### 2.3. T Cell Phenotyping

To analyze CD8+ T cell responses at the epitope level after both infection and vaccination, we selected a set of well-described and immunodominant CD8+ T cell epitopes (A*02/ORF3a_139–147_ LLYDANYFL, A*03/S_378–386_ KCYGVSPTK, B*07/N_105–113_ SPRWYFYYL and A*02/S_269–277_ YLQRPTFLL) restricted by common HLA alleles matching the HLA alleles of our myeloma patients and healthy controls. Peptides were obtained from Genaxxon Bioscience (>70% purity, amidated C terminus without N terminal modification) and loaded on HLA class I easYmers (immunAware, Hørsholm, Denmark) following the manufacturer’s instructions. Tetramers specific for the following SARS-CoV-2 epitopes were produced by conjugation of the biotinylated peptide-loaded HLA class I monomers with phycoerythrin (PE)-conjugated streptavidin (Agilent, Santa Clara, CA, USA). The median fluorescence intensity of the specific markers on SARS-CoV-2-specific CD8+ T cells was normalized to median fluorescence intensity of the specific markers on naïve bulk CD8+ T cells of the same patient.

### 2.4. In Vitro Stimulation and T Cell Functionality

A total of 1.5 × 10^6^ PBMCs were stimulated with anti-CD28 antibodies (0.5 μg mL^−1^ from BD) and the corresponding SARS-CoV-2-specific peptides (5 μM, A*02/ORF3a_139–147_ LLYDANYFL, A*03/S_378–386_ KCYGVSPTK, B*07/N_105–113_ SPRWYFYYL and A*02/S_269–277_ YLQRPTFLL) on day 0 after thawing. Cell culture medium (RPMI supplemented with 20 IU rIL-2 from StemCell Technologies, Vancouver, Canada) was changed every 3 to 4 days. T cell functionality was assessed by intracellular cytokine production and degranulation upon stimulation with spike-specific peptides (15µM) on day 14. For that, cells were stained with anti-CD107a (H4A3, 1:100 from BD Bioscience, Franklin Lakes, NJ, USA) in the first hour of restimulation at 37 °C. To augment detection of intracellular cytokines, brefeldin A (GolgiPlug, 0.5 μL mL^−1^) and monensin (GolgiStop, 0.5 μL mL^−1^, both BD Biosciences) were added to the culture for 5 h. Surface and intracellular staining were performed according to the manufacturer’s instructions (antibodies are listed in Table S2). Expansion capacity of spike-specific CD8+ T cells was performed as previously described [20]. For donors with more than one matching HLA allele, all corresponding epitopes were tested.

### 2.5. Ex Vivo Detection of Spike-Specific CD8+ T Cells

To augment the detection of rare, tetramer-reactive CD8+ T cells, enrichment of spike-specific CD8+ T cells was performed as previously described by Alanio et al. [21]. For that, 1–2 × 10^7^ PBMCs labeled with PE-conjugated peptide-loaded HLA class I tetramers (30 min at room temperature) were incubated with anti-PE beads (Milteny Biotec, Bergisch Gladbach, Germany) for 30 min at 4 °C. Magnetic enrichment (MACS) was performed according to the manufacturer’s instructions. Enriched fractions were stained for surface and intracellular markers and analyzed by flow cytometry using the LSRFortessa (BD Bioscience, Franklin Lakes, NJ, USA). Frequencies of spike-specific CD8+ T cells were calculated in all samples exceeding 5 spike-specific CD8+ T cells and were used for further phenotypic analysis. For donors with more than one matching HLA allele, all corresponding epitopes were tested. The gating strategy of flow cytometry data is depicted in Figure S1.

### 2.6. Flow Cytometric Analysis of CD8+ T Cells

After staining of surface and intracellular targets according to the manufacturer’s instructions, samples were fixed with 2% paraformaldehyde (PFA from Sigma). Data were acquired on FACSCanto II, LSRFortessa with FACSDiva software version 10.6.2 (BD) or CytoFLEX with CytExpert Software version 2.3.0.84 (Beckman Coulter, Brea, CA, USA). Gating and analysis of flow cytometry data were performed using FlowJo 10.6.2 (Treestar, San Carlos, CA, USA). All antibodies used for staining are listed in Table S2.

### 2.7. Serum IgG Determination

Anti-SARS-CoV-2 spike IgG was determined using the Euroimmun Anti-SARS-CoV-2-QuantiVac-ELISA (IgG) according to the manufacturer’s instructions (detection limit of anti-SARS-CoV-2 S IgG; <35.2 BAU mL^−1^: negative, ≥35.2 BAU mL^−1^: positive).

Detection of anti-SARS-CoV-2 N IgG was performed using the Euroimmun Mikrogen assay according to the manufacturer’s instructions (detection limit of anti-SARS-CoV-2 N IgG 24 a.u. mL^−1^).

### 2.8. Neutralization Assay

Serum samples of convalescent and vaccinated myeloma patients and healthy controls were analyzed in a plaque reduction neutralization test (PRNT) as described previously [13]. Briefly, VeroE6 cells were seeded in 12-well plates at a density of 2.8 × 10^5^ cells per well 24 h before infection. Serum sample dilutions (1:16, 1:32, 1:64, 1:128, 1:256, 1:512 and 1:1.024) were prepared in PBS with a total volume of 50 μL. A negative control was included (PBS without serum) for each sample. Diluted sera and negative controls were subsequently mixed with 90 plaque-forming units (PFUs) of either authentic SARS-CoV-2 B.1 or B.1.617 variant in 50 μL PBS (1.600 PFU mL^−1^), resulting in final sera dilution ratios of 1:32, 1:64, 1:128, 1:256, 1:512, 1:1.024 and 1:2.048. After one hour of incubation at room temperature, 400 μL of PBS was added to each sample and the resulting total mixture was used to infect VeroE6 cells. Inoculum was removed and cells were overlaid with 0.6% Oxoid-agar in DMEM, 20 mM HEPES (pH 7.4), 0.1% NaHCO3, 1% BSA and 0.01% DEAE-Dextran after 1.5 h of incubation at room temperature. Cells were fixed with 4% formaldehyde for 30 min 72 h after infection and stained with 1% crystal violet after removal of the agar overlay. PFUs were counted manually. The number of plaques counted for the serum-treated wells was compared with the average number of plaques in the untreated negative controls, which was set at 100%. The serum concentration that reduces the number of plaques by 50% was determined as the virus-neutralizing titer (PRNT50). PRNT50 values were calculated using a linear regression model in GraphPad Prism 9 (GraphPad Prism Software).

### 2.9. Statistics

Statistical analyses were conducted using the GraphPad Prism software. For the comparison of means, and unless otherwise indicated, the statistical significance of the generated data was evaluated using Mann–Whitney test, Chi^2^-test, Kruskal–Wallis or unpaired *t*-test. *p* values < 0.05 were considered to be statistically significant.

## 3. Results

### 3.1. Characteristics of COVID-19 Convalescent MM Patients and HCs

A total of 16 patients with MM and confirmed SARS-CoV-2 infection were recruited. The demographic and clinical characteristics are presented in Table 1. The median age of MM patients was 57.5 years (range 38–85), and 75% were male. MM patients presented with either mild (*n* = 7), moderate (*n* = 5) or severe (*n* = 4) COVID-19 (Figure S2A). None of the MM patients in our cohort died of COVID-19. At the time of COVID-19, 6 MM patients (37.5%) received myeloma-directed therapy, and 12 patients (75%) had received autologous hematopoietic stem cell transplantation (auto-HCT) (Table 1). The median time between auto-HCT and SARS-CoV-2 infection was 23 months (range 2–160 months). A total of 50 healthy controls were recruited at the University Hospital Freiburg. All HCs had mild or moderate COVID-19. The median age of the HCs was 50 years (range 26–88), and 47.4% were male.

### 3.2. Similar Humoral Immune Response in MM Patients and HCs After SARS-CoV-2 Infection

To investigate the humoral immune response, we analyzed spike-specific IgGs using the Euroimmun Anti-SARS-CoV-2 QuantiVac ELISA in the sera of the COVID-19 convalescent MM patients and HCs. The neutralizing capacity of SARS-CoV-2-specific antibodies against the wild-type (B.1) virus and the variant of concern (VOC) B.1.617 was tested by a plaque reduction neutralization test (PRNT).

**Figure 1:**
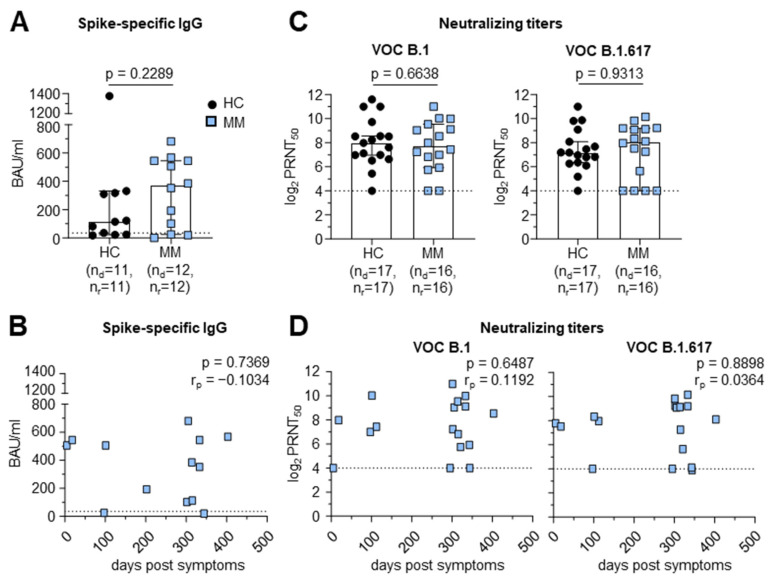
Similar humoral immune response in MM patients and HCs after SARS-CoV-2 infection. (**A**) Quantification of serum anti-SARS-CoV-2 spike immunoglobulin G (IgG) levels in MM patients versus HC. Dotted line indicates detection limit of 35.2 BAU/mL. (**B**) Longitudinal overview of spike-specific immunoglobulin G (IgG) levels in MM patients depending on the days post-symptomatic onset (detection limit: 35.2 BAU/mL). (**C**) Antibody neutralization activity as 50% plaque reduction neutralization tests (PRNT_50_) for SARS-CoV-2 variants B.1 and B.1.617. Detection limit: 4 log_2_PRNT_50_. (**D**) Longitudinal overview of antibody neutralization activity as 50% plaque reduction neutralization tests (PRNT_50_) for SARS-CoV-2 variants B.1 and B.1.617 depending on the days post-symptomatic onset. Numbers indicate non-logarithmic median value. Detection limit: 4 log_2_PRNT_50_. Statistical significance was determined by Mann–Whitney test or unpaired *t*-test comparing MM patients to HC and Pearson (r_p_) correlation. Note: MM: multiple myeloma; HC: healthy control; IgG: immunoglobulin G; VOC: variant of concern; n_d_: number of donors; n_r_: number of responses.

In contrast to data showing lower antibody levels in patients with hematologic malignancies [5], the quantification of serum anti-SARS-CoV-2 spike-specific IgG levels in MM patients (*n* = 12) versus HCs (*n* = 11) showed no significant differences in the IgG levels in the two cohorts (median 368.4 BAU/mL in MM vs. 114.1 BAU/mL in HCs, *p* = 0.2289) (Figure 1A) without detectable waning up to 400 days after symptom onset (Figure 1B). Consistent with this observation, the levels of neutralizing antibodies against B1 (median log2 PRNT50: 7.493 in MM vs. 8.024 in HCs, *p* = 0.4689) and VOC B1.617 (median log2 PRNT50: 7.367 in MM vs. 7.402 in HCs, *p* = 0.9598) did not significantly differ in our cohort of MM patients (*n* = 16) compared to the control group (*n* = 17) (Figure 1C) irrespective of the day of post-symptom onset (Figure 1D).

In summary, convalescent MM patients elicited similar spike-specific IgG levels and neutralizing antibody levels against SARS-CoV-2 as the HCs.

### 3.3. Impaired Virus-Specific CD8+ T Cell Response in MM Patients Following SARS-CoV-2 Infection

We collected peripheral blood mononuclear cells (PBMCs) from patients with MM and HCs with a history of COVID-19 (Table S1). To determine the induction of SARS-CoV-2-specific CD8+ T cell immunity, we stimulated the PBMCs with peptides of the well-described SARS-CoV-2-specific CD8+ T cell epitopes A*02/ORF3a_139–147_ (LLYDANYFL), A*03/S_378–386_ (KCYGVSPTK) and B*07/N_105–113_ (SPRWYFYYL). Accordingly, PBMCs from 13 MM patients and 43 HCs expressing the respective HLA alleles were tested for SARS-CoV-2-specific CD8+ T cell responses.

**Figure 2:**
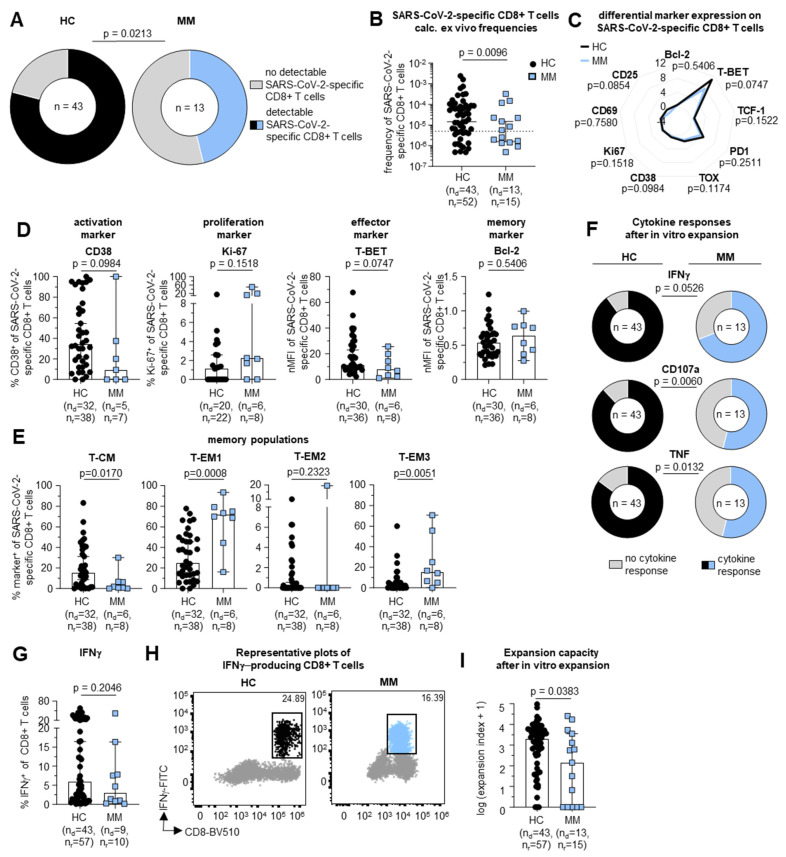
Impaired virus-specific CD8+ T cell response in MM patients following SARS-CoV-2 infection. (**A**) Tested MM patients (blue) or HC (black) expressing either HLA-A*02, -A*03 or B*07 with and without ex vivo detectable SARS-CoV-2-specific CD8+ T cells. (**B**) Calculated ex vivo frequencies of SARS-CoV-2-specific CD8+ T cells in MM patients (blue) versus healthy controls (black). Dotted line indicates detection limit of 5 × 10^−6^. (**C**) Spider plot with differential marker expression, as nMFI, on SARS-CoV-2-specific non-naïve CD8+ T cells in MM patients. (**D**) Expression levels of representative markers for activation (CD38), proliferation (Ki-67), effector function (T-BET) and memory (Bcl-2) in MM patients (blue) versus HC (black). (**E**) Distribution of SARS-CoV-2-specific CD8+ T cell memory subsets T-CM, T-EM1, T-EM2 and T-EM3 in MM patients versus HC. (**F**) Number of IFNγ, CD107a or TNF positive and negative responses after 14 days of in vitro expansion in MM patients versus HC. (**G**) Percentage of IFNγ-producing CD8+ T cells related to all CD8+ T cells after in vitro expansion. (**H**) Representative plots for IFN-γ-producing CD8+ T cells in MM patients and HC. (**I**) Expansion capacity of SARS-CoV-2-specific CD8+ T cells over 14 days of in vitro expansion in MM patients vs. HC. Median values are depicted with 95% confidence interval error bars. Statistical significance was determined by Chi^2^-test, Mann–Whitney test or unpaired *t*-test comparing MM patients to HC. Note: nMFI: normalized median fluorescence intensity; MM: multiple myeloma; HC: healthy control; T-CM: central memory T cells; T-EM: effector memory T cellsn_d_: number of donors; n_r_: number of responses.

Among MM patients, the overall proportion of individuals with ex vivo detectable SARS-CoV-2-specific CD8+ T cells was significantly reduced compared to the HCs (6/13 [46%] in MM patients vs. 34/43 [79%] in HCs, *p* = 0.0213) (Figure 2A). Additionally, the frequencies of ex vivo detectable SARS-CoV-2-specific CD8+ T cells were significantly reduced in patients with MM compared to the HCs (1.57 × 10^−6^ vs. 1.43 × 10^−5^, respectively, *p* = 0.0096) (Figure 2B). The CD8+ T cell frequencies in MM patients were constant irrespective of the day of post-symptom onset (Figure S2B). Furthermore, we did not observe differences in the frequencies of detectable SARS-CoV-2-specific CD8+ T cells when comparing the different subtypes of MM (IgG lambda, IgG kappa, IgA kappa and kappa LC) (Figure S2C, representative plots in Figure S2D).

Next, we comparatively investigated the phenotype of SARS-CoV-2-specific CD8+ T cells in MM patients. Specifically, we analyzed the expression of activation, differentiation and memory markers following peptide/MHC class I tetramer enrichment of SARS-CoV-2-specific CD8+ T cells in both cohorts. By this approach, we observed a similar phenotypic imprint of SARS-CoV-2-specific CD8+ T cells in MM patients versus HCs (Figure 2C). For example, we detected a comparable expression of markers for activation (CD38, *p* = 0.0984), proliferation (Ki-67, *p* = 0.1518), effector function (T-BET, *p* = 0.0747) and memory (Bcl-2, *p* = 0.5406) (Figure 2D) in both cohorts. As expected, SARS-CoV-2-specific T cell activation decreased longitudinally following SARS-CoV-2 infection (Figure S2E). Interestingly, we observed differences in the memory T cell subset distribution of SARS-CoV-2-specific CD8+ T cells with lower proportions of more early differentiated central memory (T_CM_) T cell subsets (defined as CD45RA−, CCR7+ and CD27+) as well as higher frequencies of effector memory 1 T cells (T_EM1_) (defined as CD45RA−, CCR7− and CD27+) and effector memory 3 T cells (T_EM3_) (defined as CD45RA−, CCR7− and CD27−) in MM patients (Figure 2E).

Finally, we set out to evaluate the functional properties of SARS-CoV-2-specific CD8+ T cells in MM patients in comparison to HCs. In particular, when comparing donors with a detectable CD8+ T cell response, the proportion of virus-specific CD8+ T cells with measurable degranulation (CD107a: 7/13 [53.85%] in MM patients vs. 38/43 [88.37%] in HCs, *p* = 0.0060) and cytokine production (IFNγ: 9/13 [69.23%] in MM patients vs. 39/43 [90.69%] in HCs, *p* = 0.0526; TNF: 7/13 [53.85%] in MM patients vs. 37/43 [85.04%] in HCs, *p* = 0.0132) was lower in the MM patients in comparison to HCs (Figure 2F). However, when comparing CD8+ T cells that show degranulation or cytokine production, we observed similar levels of IFNγ (*p* = 0.2046) (Figure 2G, representative plots in Figure 2H), TNF (*p* = 0.2500) and CD107a (*p* = 0.2732) (Figure S2F). We also determined the expansion capacity after in vitro stimulation for 14 days in HCs (*n* = 43) and MM patients (*n* = 13). In line with the degranulation and cytokine production-based T cell response, we observed a significantly weaker expansion capacity of SARS-CoV-2-specific CD8+ T cells obtained from MM patients (expansion index (log^+1^) 2.154 in MM patients vs. 3.314 in HCs, *p* = 0.0383) (Figure 2I).

Collectively, the frequencies of virus-specific CD8+ T cells are reduced in MM patients following SARS-CoV-2 infection and the detectable CD8+ T cells display a distinct memory subset distribution as well as reduced expansion capacity when compared to the HCs.

### 3.4. Impaired mRNA Vaccine-Induced Cellular Immune Response in MM Patients

Next, we set out to investigate vaccine-induced CD8+ T cell immunity after mRNA vaccination in patients with MM without prior SARS-CoV-2 infection (based on reporting and anti-nucleocapsid IgG testing). With respect to the currently recommended basic immunity against SARS-CoV-2 induced by three antigen contacts, e.g., by three doses of an mRNA vaccine, we collected corresponding samples from fully vaccinated MM patients (*n* = 5) and HCs (*n* = 7) (Table 2 and Table S1).

**Figure 3:**
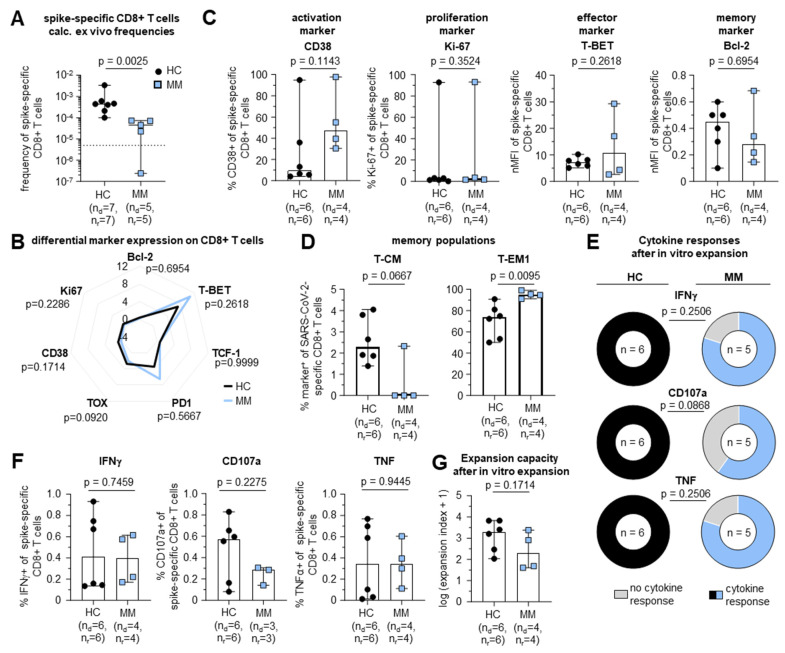
Impaired mRNA vaccine-induced cellular immune response in MM patients. (**A**) Calculated ex vivo frequencies of SARS-CoV-2 spike-specific CD8+ T cells in MM patients (blue) versus HC (black) (dotted line indicates detection limit of 5 × 10^−6^). (**B**) Spider plots with differential marker expression, as nMFI, on SARS-CoV-2-specific non-naïve CD8+ T cells in MM patients versus HC. (**C**) Expression levels of CD38 and Ki-67 within SARS-CoV-2-specific non-naïve CD8+ T cells and nMFI (normalized to naïve CD8+ T cells) of T-BET and Bcl-2 in MM patients (blue) versus HC (black). (**D**) Distribution of SARS-CoV-2-specific CD8+ T cell memory subsets T-CM and T-EM1 in MM patients versus HC. (**E**) Overall number of IFNγ, CD107a or TNF positive and negative responses after 14 days of in vitro expansion in MM patients versus HC. (**F**) Percentage of IFNγ−, CD107a− or TNF-producing CD8+ T cells related to all CD8+ T cells after in vitro expansion. (**G**) Expansion capacity of SARS-CoV-2 spike-specific CD8+ T cells over 14 days of in vitro expansion. Median values are depicted with 95% confidence interval error bars. Statistical significance was determined by Mann–Whitney test or unpaired *t*-test comparing MM patients to HC. Note: nMFI: normalized median fluorescence intensity; MM: multiple myeloma; HC: healthy control; n_d_: number of donors; n_r_: number of responses.

Similar to the observation of reduced frequencies of SARS-CoV-2-specific CD8+ T cells in convalescent MM patients, three mRNA vaccine doses also induced significantly lower frequencies of spike-specific CD8+ T cells against the T cell epitope A*02/S_269-277_ in MM patients compared to HCs (4.520 × 10^−5^ vs. 4.450 × 10^−4^, *p* = 0.0025) (Figure 3A). The phenotypic imprint of the vaccine-induced CD8+ T cell response was comparable in MM patients and HCs (Figure 3B). Indeed, we observed a similar marker expression including markers for activation and effector function, such as CD38 (*p* = 0.1143) or T-BET (*p* = 0.2618) (Figure 3C). Regarding the CD8+ T cell memory differentiation, we again observed lower proportions of early differentiated T_CM_ cell subsets and higher frequencies of T_EM1_ cells in MM patients compared to HCs (Figure 3D).

To assess the functionality of spike-specific CD8+ T cells in fully vaccinated MM patients, we analyzed the degranulation and cytokine production. As shown for natural infection, donors with a detectable CD8+ T cell response displayed a lower proportion of CD8+ T cells with measurable cytokine production (IFNγ: 4/5 [80%] in MM patients vs. 6/6 [100%] in HCs, *p* = 0.2506; TNF: 4/5 [80%] in MM patients vs. 6/6 [100%] in HCs, *p* = 0.2506) and degranulation (CD107a: 3/5 [60%] in MM patients vs. 6/6 [100%] in HCs, *p* = 0.0868) (Figure 3E).

In addition, the levels of cytokine production and degranulation were similar in MM patients with detectable spike-specific CD8+ T cells compared to HCs (IFNγ: 0.40 in MM patients vs. 0.41 in HCs (*p* = 0.7459); TNF: 0.35 in MM patients vs. 0.35 in HCs (*p* = 0.9445); CD107a: 0.28 in MM patients vs. 0.57 in HCs (*p* = 0.2275)) (Figure 3F). In contrast to natural SARS-CoV-2 infection, where expansion capacity was impaired in MM patients, we observed a similar expansion capacity after 14 days of in vitro stimulation in vaccinated MM patients compared to HCs (Figure 3G).

In conclusion, the frequency of vaccine-induced CD8+ T cells targeting the spike protein and the proportion of functional CD8+ T cells were reduced in vaccinated MM patients compared to HCs after basic immunization, whereas the expansion capacity of CD8+ T cells was similar in both cohorts. Furthermore, as in SARS-CoV-2 infection, the phenotype of spike-specific CD8+ T cells showed differences in the memory T cell subsets.

### 3.5. Robust Hybrid Immune Activation in MM Patients After COVID-19 mRNA Vaccination

To test hybrid immune activation in MM patients, we investigated the impact of mRNA vaccination (either BNT162b2 or mRNA-1273) on the adaptive immune response in COVID-19 convalescent MM patients (Table S1).

**Figure 4:**
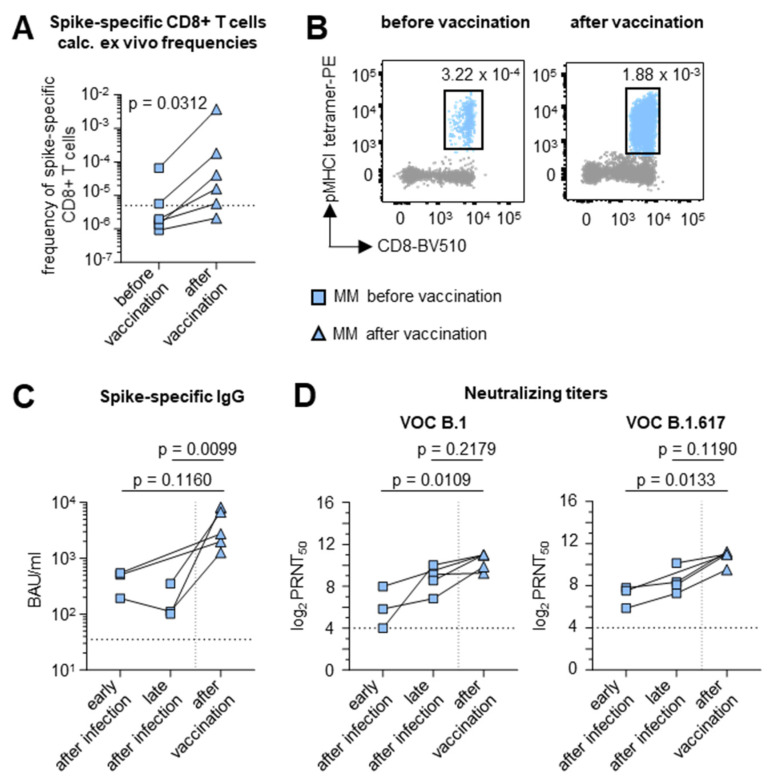
Robust hybrid immune activation in MM patients after COVID-19 mRNA vaccination. (**A**) Calculated ex vivo frequencies of SARS-CoV-2 spike-specific CD8+ T cells in MM patients before (rectangle) and after (triangle) first SARS-CoV-2 boost vaccination. Detection limit: 5 × 10^−6^. (**B**) Representative tetramer stainings of SARS-CoV-2-specific CD8+ T cells in one patient before and after first SARS-CoV-2 boost vaccination. (**C**) Quantification of serum anti-SARS-CoV-2 spike IgG levels early (1–230 dps), late (250–410 dps) and after first SARS-CoV-2 boost vaccination. Detection limit: 35.2 BAU/mL. (**D**) Antibody neutralization activity, as 50% plaque reduction neutralization tests (PRNT50) for SARS-CoV-2 variants B.1 and B.1.617, early (1–230 dps), late (250–410 dps) and after first SARS-CoV-2 boost vaccination. Numbers indicate non-logarithmic median value. Detection limit: 4 log2PRNT50. Kruskal–Wallis test was used comparing the time course effect. Note: MM: multiple myeloma; IgG: immunoglobulin G; VOC: variant of concern.

After stimulating PBMCs from HLA-A*02-positive MM patients (*n* = 3) with the peptide corresponding to the immunodominant spike-specific CD8+ T cell epitope A*02/S_269–277_ (YLQRPTFLL), we compared the ex vivo frequencies of spike-specific CD8+ T cells before and after SARS-CoV-2 mRNA vaccination. A single shot of the mRNA vaccine induced an increase in the frequency of SARS-CoV-2-specific CD8+ T cells in all three tested individuals (Figure 4A,B).

The administration of a single mRNA vaccine dose also enhanced the humoral immune response against SARS-CoV-2. In particular, the ELISA-based analysis of spike-specific IgGs revealed significantly increasing antibody levels in convalescent MM patients (*n* = 5, *p* = 0.0099) (Figure 4C). In line with this, the levels of neutralizing antibodies against the B.1 and B.1.617 variants increased in the same MM patients following mRNA vaccination (*p* = 0.0109 and *p* = 0.0133, respectively) (Figure 4D).

In summary, these data suggest that hybrid immunity is able to at least partially compensate for the impaired adaptive immune response in MM patients.

## 4. Discussion

The immune system of MM patients is dysregulated due to a plasma cell infiltration of the bone marrow and immunosuppressive myeloma-directed therapies, resulting in an increased susceptibility to infection [1,2,3]. Therefore, prophylactic vaccinations may be particularly beneficial in this vulnerable patient population to manage severe infection courses. However, humoral and cellular immune responses induced by both infection and vaccination in MM patients are poorly characterized. The emergence of the SARS-CoV-2 pandemic in 2019 and the rapid development of vaccines represent unique opportunities to analyze immunity to infection and vaccination in MM patients. In this study, we therefore assessed SARS-CoV-2-related immunity to provide a comprehensive analysis of the impact of both infection and vaccination on the adaptive immune response in MM patients. In addition to determining the virus-specific humoral immune response, we performed, to our knowledge, the first in-depth characterization of SARS-CoV-2-specific CD8+ T cell immunity at the epitope level in COVID-19 convalescent and mRNA-vaccinated MM patients.

With regard to the humoral immune response, our study revealed robust antibody responses with high titers of spike-specific IgGs and neutralizing antibodies against B1 and B1.617 variants comparable to HCs. This observation is consistent with an earlier study in COVID-19 convalescent MM patients that also reported adequate levels of virus-specific IgGs and neutralizing antibodies [22]. While a successful autologous stem cell transplantation can lead to a reconstitution of the humoral immune response, other MM therapies are associated with an impaired antibody response [23,24,25]. The robust antibody responses in our cohort of MM patients may thus be explained by a high remission rate of 31.3% and a high proportion of patients without requirement of myeloma-directed therapy (62.7%) at the time of COVID-19 (Table 1). Of note, limited sample availability restricted testing of the neutralization capacity against the Omicron VOC in this study. However, a recent study by Keppler-Hafkemeyer et al. described relevant titers of neutralizing antibodies to the Omicron VOC after vaccination of MM patients [26].

In addition to the humoral immune response, virus-specific CD8+ T cells constitute an important component of the adaptive immune response to SARS-CoV-2. Although SARS-CoV-2-specific CD8+ T cells have been characterized in detail in immunocompetent individuals [12,13,14,15,16,17,18,27,28,29,30], SARS-CoV-2-specific CD8+ T cell-mediated immunity has remained poorly described in convalescent or vaccinated MM patients. Available studies investigating virus-specific CD8+ T cells use a quantitative approach with SARS-CoV-2 peptide pools to assess virus-specific T cell responses [5,6,26,31]. In this study, we extended this knowledge to an in-depth phenotypic and functional characterization of epitope-specific CD8+ T cells induced by either SARS-CoV-2 infection or vaccination. We observed that the frequencies of detectable virus-specific CD8+ T cells and the proportion of fully functional CD8+ T cells was lower in COVID-19 convalescent MM patients compared to HCs. This impaired CD8+ T cell-mediated immune response may partially explain the higher risk of severe COVID-19 in MM patients [8], since strong virus-specific CD8+ T cell responses are known to be associated with a mild course of infection [32].

The EPICOVIDEHA registry study [8] described significantly higher overall survival in vaccinated MM patients. In order to determine the vaccine-elicited CD8+ T cell response, we examined in this study the influence of three doses of the mRNA vaccine on virus-specific CD8+ T cells in MM patients without prior history of SARS-CoV-2 infection. Frequencies of virus-specific CD8+ T cells were lower in fully vaccinated MM patients compared to HCs. However, the proportion of spike-specific CD8+ T cells showing cytokine production and degranulation was higher than after natural infection, but still lower when compared to HCs. These observations are in line with and extend previous reports showing the induction of spike-specific CD8+ T cells in fully vaccinated MM patients [6,26,33]. Yet, further studies are needed to investigate the impact of additional booster vaccinations on the frequency and function of spike-specific CD8+ T cells in MM patients to ameliorate the course of SARS-CoV-2 infection in these vulnerable patients. However, antibody responses following mRNA vaccination have been investigated more comprehensively in diverse studies, e.g., in the study by Storti et al. [34], showing that MM patients mount virus-specific IgG and neutralizing antibodies against all common VOCs after vaccination. Similar to healthy individuals [27], antibody titers against the currently predominant Omicron VOC were reduced in MM patients, but can be improved by booster mRNA vaccination [34]. Combined, these observations show that mRNA vaccination elicits an adaptive immune response covering the humoral and the CD8+ T cell arm in MM patients.

By analyzing the CD8+ T cell response on the single-epitope level, we were able to deeply characterize CD8+ T cell memory formation in MM patients after mRNA vaccination. We observed differences in the spike-specific CD8+ T cell memory subset distribution in MM patients in comparison to HCs. After both natural infection and complete mRNA vaccination, we detected lower frequencies of early differentiated central memory (T_CM_) T cell subsets (defined as CD45RA−, CCR7+ and CD27+) as well as higher frequencies of effector memory 1 T cells (T_EM1_) (defined as CD45RA−, CCR7− and CD27+) in MM patients. In convalescent MM patients, we also observed higher proportions of effector memory 3 T cells (T_EM3_) (defined as CD45RA−, CCR7− and CD27−). T_CM_ and T_EM_ cells differ in the expression of CCR7, leading to different migratory characteristics of these memory cells [35,36]. While CCR+ T_CM_ circulate through and are localized in secondary lymph nodes, CCR7- T_EM_ are primarily found in blood and peripheral non-lymphoid tissues [37]. TCM cells incorporate the capacity for self-renewal with long-term persistence and the ability to expand rapidly when encountering the antigen, while TEM cells have higher cytotoxic efficacy and are ready to elicit effector functions [38,39,40,41]. One reason for this altered subset distribution may be the different activation strength and dynamics in MM. Hence, the longevity of T_CM_ is important to ensure long-lasting protection against pathogens both after infection and after vaccination. Thus, our observation of decreased frequencies of T_CM_ in MM patients compared to HCs may potentially indicate an impairment of long-term immune protection against SARS-CoV-2 in the immunocompromised MM patients. As a consequence, further studies are needed to evaluate the long-term immune response and the influence of repeated booster vaccinations in these patients to assess whether they would benefit from a stricter vaccination regimen compared to HCs.

Finally, we set out to determine whether a single mRNA vaccine dose may improve the adaptive immune response in convalescent MM patients. Interestingly, we observed an improvement in both the CD8+ T cell frequencies and the antibody response in the MM patients. This superior infection-plus-vaccination-induced hybrid immunity has also previously been described in immunocompetent individuals [42,43,44] and patients with solid cancer or cirrhosis [6,45], highlighting the importance of adjunctive vaccinations after SARS-CoV-2 infection in vulnerable patient cohorts including patients with hematologic malignancies.

A limitation of this study is the small sample size of the MM cohort, which hinders our ability to analyze the immune responses in a larger cohort and to explore the influence of different myeloma-directed treatment modalities on humoral and CD8+ T cell responses. A further limitation of this study is the lack of samples from single-vaccinated HCs with a history of SARS-CoV-2 infection, which prevents us from comparing the hybrid immunity in MM patients to that of HC. For the same reason, we were also not able to compare vaccinated convalescent MM patients with MM patients infected with SARS-CoV-2 but not vaccinated.

In conclusion, our study underpins the vulnerability of MM patients with regard to viral infections since virus-specific CD8+ T cell responses are impaired. However, hybrid immune activation can at least partly compensate for this adaptive immune impairment, highlighting the relevance of vaccinating convalescent patients of vulnerable groups.

## Figures and Tables

**Table 1:** Baseline patient characteristics of the convalescent MM cohort.

Variable	Value
Total number of patients	16
Median age (range) at time of COVID-19—years	57.5 (38–85)
Sex—no. (%)	
- Female - Male	4 (25%) 12 (75%)
MM type—no. (%)	
- IgG kappa - IgG lambda - IgA kappa - Kappa LC	9 (56.3%) 4 (25%) 1 (6.2.%) 2 (12.5%)
MM cytogenetic risk classification—no. (%)	
- Standard risk - High risk - Unknown	9 (56.3%) 5 (31.2%) 2 (12.5%)
Staging at diagnosis of MM	
- SMM - ISS I - ISS II - ISS III	1 (6.2%) 7 (43.8%) 7 (43.8%) 1 (6.2%)
Auto-HCT—no. (%)	
- Yes - No	12 (75%) 4 (25%)
Prior lines of therapy at time of COVID-19	
- 0 - 1 - 2 - 3 - 4 - 5	3 (18.8%) 9 (56.3%) 1 (6.2%) 1 (6.2%) 1 (6.2%) 1 (6.2%)
Therapy at time of COVID-19	
- Watch and wait - Lenalidomide maintenance - Bortezomib/Cyclophosphamide - Daratumumab/Bortezomib/Dexamethasone - Daratumumab/Lenalidomide/Dexamethasone - Daratumumab/Ixazomib/Dexamethasone	10 (62.7%) 2 (12.5%) 1 (6.2%) 1 (6.2%) 1 (6.2%) 1 (6.2%)
Disease status at time of COVID-19	
- Initial diagnosis - CR - VGPR - PR - SD - PD	2 (12.5%) 5 (31.3%) 4 (25%) 1 (6.2%) 3 (18.8%) 1 (6.2%)
Charlson Comorbidity Index at time of COVID-19	
- 2 - 3 - 4 - 5 - 6 - 9	1 (6.2%) 6 (37.5%) 3 (18.8%) 2 (12.5%) 3 (18.8%) 1 (6.2%)

Abbreviations: Auto-HCT, autologous hematopoietic cell transplantation; CR, complete remission; ISS, International Staging System; LC, light chain; MM, multiple myeloma; SD, stable disease; SMM, smoldering multiple myeloma; PD, progressive disease; PR, partial remission; VGPR, very good partial remission.

**Table 2:** Baseline patient characteristics of MM vaccine cohort.

Variable	Value
Total number of patients	5
Median age (range) at time of vaccination—years	75 (54–78)
Sex—no. (%)	
- Female - Male	4 (80%) 1 (20%)
MM type—no. (%)	
- IgG kappa - IgG lambda	4 (80%) 1 (20%)
MM cytogenetic risk classification—no. (%)	
- Standard risk - High risk - Unknown	3 (60%) 1 (20%) 1 (20%)
Staging at diagnosis of MM	
- ISS I - ISS II - ISS III	2 (40%) 2 (40%) 1 (20%)
Auto-HCT—no. (%)	
- Yes - No	3 (60%) 2 (40%)
Therapy at time of vaccination	
- Watch and wait - Lenalidomide maintenance	1 (20%) 4 (80%)
Disease status at time of vaccination	
- CR - VGPR - PR - SD	1 (20%) 1 (20%) 2 (40%) 1 (20%)
Charlson Comorbidity Index at time of vaccination	
- 3 - 4 - 5 - 8	1 (20%) 1 (20%) 2 (40%) 1 (20%)

Auto-HCT, autologous hematopoietic cell transplantation; CR, complete remission; ISS, International Staging System; MM, multiple myeloma; SD, stable disease; PR, partial remission; VGPR, very good partial remission.

## Data Availability

The datasets used and/or analyzed in this study are available upon reasonable request from the corresponding author.

## Supplementary Materials

The following supporting information can be downloaded at: https://www.mdpi.com/article/10.3390/vaccines12111249/s1, Figure S1: gating strategy of flow cytometry data; Figure S2: COVID-19 disease severity, analyzed spike-specific epitopes, representative plots and longitudinal representation of SARS-CoV-2-specific immune responses in MM patients; Table S1: donors’ characteristics; Table S2: antibodies used for multiparametric flow cytometry.
