# Supplementary material for: Impaired SARS-CoV-2-Specific CD8+ T Cells After Infection or Vaccination but Robust Hybrid T Cell Immunity in Patients with Multiple Myeloma
Source: Vaccines (Basel). 2024 Nov 1;12(11):1249. doi: 10.3390/vaccines12111249 (PMC11598869; doi:10.3390/vaccines12111249)
Supplement: Supplementary file 1 [file vaccines-12-01249-s001.zip › vaccines-3250206-supplementary.pdf]

Supplementary Materials

Figure S1. Gating strategy of flow cytometry data

Figure S2. COVID-19 disease severity, analyzed spike-specific epitopes, representative plots and longitudinal representation of SARS-CoV-2-specific immune responses in MM patients.

Table S1. Donors' characteristics.

Table S2. Antibodies used for multiparametric flow cytometry

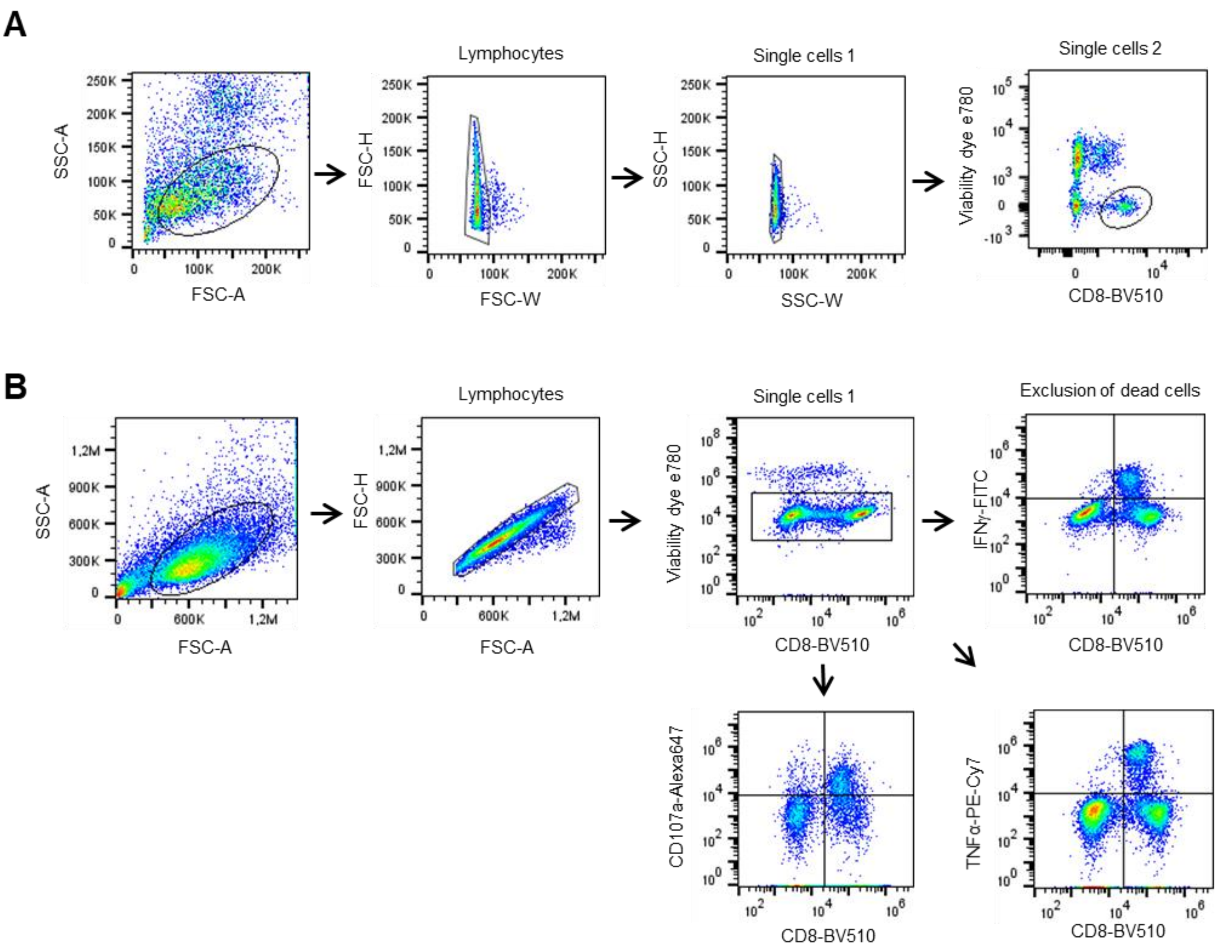

Figure S1: Gating strategy of flow cytometry data

(A) Gating strategy of tetramer-based data. A*02/ORF3a$_{139\text{-}147}$-, A*02/S$_{269\text{-}277}$-, A*03/S$_{378\text{-}386}$-, B*07/N$_{105\text{-}113}$-specific CD8+ T cells. Lymphocytes were identified via pMHCI tetramer-based analysis. (B) Gating strategy of intracellular cytokine staining. Lymphocytes were gated on FSC-A and SSC-A, doublet exclusion on FSC-H and FSC-W, exclusion of dead cells, gating on CD8+ cells.

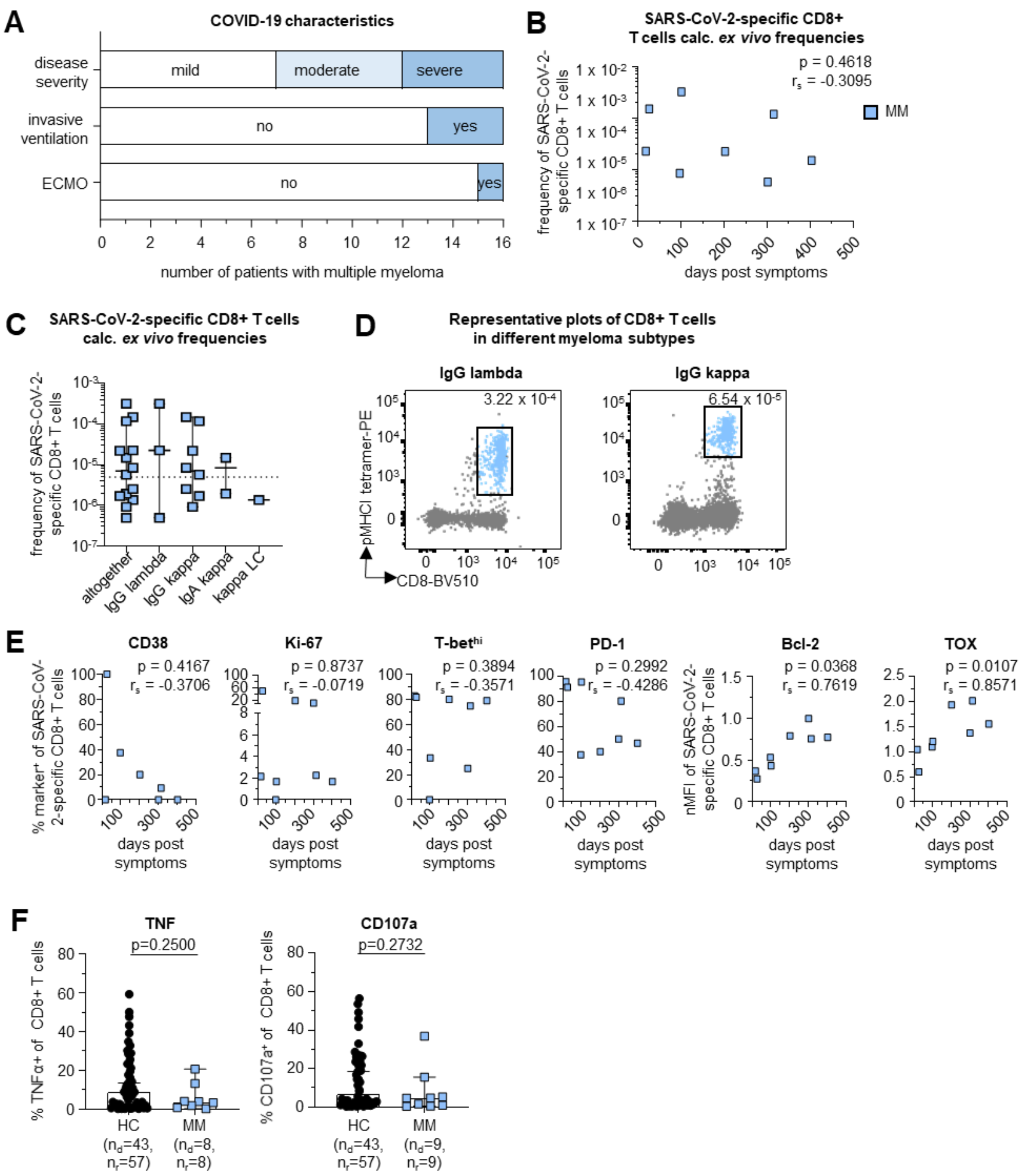

Figure S2: COVID-19 disease severity, analyzed spike-specific epitopes, representative plots and longitudinal representation of SARS-CoV-2-specific immune responses in MM patients.

(A) Description of COVID-19 disease severity according to WHO classification. (B) Longitudinal overview of calculated *ex vivo* frequencies of SARS-CoV-2-specific CD8+ T cells in MM patients depending on the days post symptomatic onset. (C) Calculated *ex vivo* frequencies of SARS-CoV-2-specific CD8+ T cells in MM patients according to the myeloma subtype. (D) Representative plots of CD8+ T cells in different myeloma subtypes. (E) Longitudinal overview of representative markers for activation (CD38 and PD-1), proliferation (Ki-67), effector function (T-bet) and memory (Bcl-2) in MM patients depending on the days post symptomatic onset. (F) Percentage of TNF- and CD107a-producing CD8+ T cells related to all CD8+ T cells after *in vitro* expansion.

Median values are depicted with 95% confidence interval error bars. Median values are depicted with 95% confidence interval error bars. Statistical significance was determined by Kruskal-Wallis test comparing MM to HC and Spearman (r_s) correlation.

MM: multiple myeloma; HC: healthy control; ECMO: Extracorporeal membrane oxygenation; Ig: Immunoglobulin; n_d*: number of donors;* n_r*: number of responses*

Table S1. Donors' characteristics.

ID	Age	Sex	HLA-A	HLA-A	HLA-B	HLA-B	T cell analyses	Spike-IgG ELISA	neutralizing antibodies	SARS-CoV-2 infection	number of mRNA vaccinations
MM1	63	m	02:01	03:01	18:01	35:03	yes	no	yes	yes	-
MM2	60	m	03:01	66:01	41:01	51:01	yes	yes	yes	yes	1
MM3	71	f	03:01	03:02	37:01	51:01	yes	yes	yes	yes	1
MM4	57	m	26:01	68:02	15:01	50:01	no	no	yes	yes	-
MM5	56	m	02:01	24:02	35:01	35:01	yes	yes	yes	yes	-
MM6	60	f	02:06	24:06	46:01	51:01	yes	yes	yes	yes	-
MM7	59	m	01:01	02:01	08:01	14:01	yes	yes	yes	yes	1
MM8	57	m	01:01	02:01	08:01	27:05	yes	yes	yes	yes	-
MM9	51	m	24:02	32:01	35:01	51:01	no	no	no	yes	-
MM10	85	f	02:01	11:01	07:01	13:02	yes	yes	yes	yes	-
MM11	38	m	32:01	68:01	13:03	44:03	no	no	yes	yes	-
MM12	54	m	03:01	03:01	07:02	07:02	yes	yes	yes	yes	-
MM13	52	m	02:01	24:01	07:02	40:01	yes	yes	yes	yes	1
MM14	74	f	02:01	29:01	07:02	15:01	yes	yes	yes	yes	1
MM15	55	m	02:01	29:01	18:01	35:02	yes	yes	yes	yes	-
MM16	67	m	02:01	03:01	07:02	51:01	yes	yes	yes	yes	-
MM17	61	f	02:01	03:01	51:01	51:01	yes	no	no	no	3
MM18	54	m	02:01	26:01	14:02	27:05	yes	no	no	no	3
MM19	75	f	01:01	02:01	07:02	27:05	yes	no	no	no	3
MM20	76	f	02:01	23:01	27:05	35:01	yes	no	no	no	3
MM21	78	f	02:01	23:01	13:02	44:03	yes	no	no	no	3
HC1	49	f	03:01	33:01	15:01	38:01	yes	no	yes	yes	-
HC2	73	f	03:01	03:01	35:01	40:02	yes	yes	yes	yes	-
HC3	72	f	02:01	03:01	15:01	15:01	yes	no	yes	yes	-
HC4	58	m	02:01	68:01	07:02	44:02	yes	no	yes	yes	-
HC5	28	m	02:01	31:01	07:02	35:01	yes	yes	yes	yes	-
HC6	38	f	02:01	03:01	27:02	57:01	yes	yes	yes	yes	-
HC7	64	m	01:01	02:01	07:02	18:01	yes	yes	yes	yes	-
HC8	52	f	03:01	11:01	07:02	08:01	yes	yes	yes	yes	-
HC9	39	f	03:01	03:01	07:02	35:01	yes	yes	yes	yes	-
HC10	32	f	02:01	02:01	18:01	44:02	yes	yes	yes	yes	-
HC11	57	m	02:01	68:01	13:02	51:01	yes	yes	yes	yes	-
HC12	56	m	02:01	11:01	27:02	55:01	yes	yes	yes	yes	-
HC13	69	f	03:01	29:95	07:02	44:03	yes	yes	yes	yes	-
HC14	43	m	03:01	29:02	40:02	44:03	yes	yes	yes	yes	-
HC15	62	f	02:01	02:01	07:02	44:02	yes	no	no	no	3
HC16	32	f	01:01	02:01	07:02	37:01	yes	no	no	no	3
HC17	33	m	02:01	02:01	15:01	51:01	yes	no	no	no	3
HC18	64	m	01:01	02:01	08:01	15:01	yes	no	no	no	3
HC19	52	f	02:01	24:02	27:05	27:07	yes	no	no	no	3
HC20	34	m	02:01	11:01	15:01	35:01	yes	no	no	no	3
HC21	37	m	02:01	68:01	15:01	51:01	yes	no	no	no	3
HC22	34	m	02:01	26:01	07:02	44:01	yes	no	no	yes	-
HC23	48	f	02:01	11:01	07:02	49:01	yes	no	no	yes	-
HC24	33	f	02:01	02:01	18:01	38:01	yes	no	no	yes	-
HC25	34	m	02:01	24:02	38:01	44:03	yes	no	no	yes	-
HC26	62	f	02:01	24:02	27:05	55:01	yes	no	no	yes	-
HC27	31	m	01:01	02:01	15:01	51:01	yes	no	no	yes	-
HC28	30	f	02:01	24:02	39:01	51:01	yes	no	no	yes	-

HC29	38	m	02:01	02:01	18:01	39:24	yes	no	no	yes	-
HC30	28	m	02:01	31:01	07:02	15:01	yes	no	no	yes	-
HC31	26	f	02:01	23:01	40:01	50:01	yes	no	no	yes	-
HC32	33	f	02:01	03:01	40:01	44:02	yes	no	no	yes	-
HC33	38	m	01:01	29:01	07:02	40:01	yes	no	no	yes	-
HC34	88	f	02:01	31:01	13:02	40:01	yes	no	no	yes	-
HC35	64	f	02:01	32:01	27:05	51:01	yes	no	no	yes	-
HC36	50	f	02:01	03:01	07:02	13:02	yes	no	no	yes	-
HC37	36	m	03:01	24:02	44:03	45:01	yes	no	no	yes	-
HC38	43	f	03:01	11:01	15:01	35:01	yes	no	no	yes	-
HC39	34	f	03:01	26:08	15:01	35:03	yes	no	no	yes	-
HC40	54	m	03:01	24:02	08:01	39:06	yes	no	no	yes	-
HC41	31	m	03:01	24:02	07:02	44:02	yes	no	no	yes	-
HC42	58	f	03:01	34:02	07:02	35:03	yes	no	no	yes	-
HC43	57	m	03:01	03:01	07:02	35:01	yes	no	no	yes	-
HC44	55	m	03:01	11:01	07:02	08:01	yes	no	no	yes	-
HC45	54	m	03:01	30:06	07:02	51:01	yes	no	no	yes	-
HC46	54	m	01:01	03:01	07:02	08:01	yes	no	no	yes	-
HC47	56	f	02:01	03:01	15:01	18:01	yes	no	no	yes	-
HC48	46	f	02:01	03:01	07:02	35:08	yes	no	no	yes	-
HC49	50	m	01:01	02:01	35:02	57:01	yes	no	no	yes	-
HC50	57	m	01:01	03:01	08:01	35:01	yes	no	no	yes	-
HC51	60	f	01:01	02:01	08:01	15:01	yes	no	no	yes	-
HC52	40	f	03:01	25:01	07:02	44:02	yes	no	no	yes	-
HC53	65	f	03:01	25:01	07:02	18:01	yes	no	no	yes	-
HC54	60	f	03:01	32:01	35:01	35:01	yes	no	no	yes	-
HC55	41	m	01:01	02:01	08:01	52:01	yes	no	no	yes	-
HC56	44	m	03:01	68:01	08:01	38:01	no	no	yes	yes	-
HC57	28	f	03:01	30:04	50:01	51:01	no	no	yes	yes	-
HC58	56	f	01:01	01:01	07:02	40:01	no	no	yes	yes	-

ID, identification name; MM, multiple myeloma; HC, healthy control; m, male; f, female;
IgG, immunoglobulin G

Table S2. Antibodies used for multiparametric flow cytometry

Company	Antibody
BD Biosciences:	anti-CCR7-PE-CF594 (150503, 1:50), Cat# 353232<br>anti-CCR7-BUV395 (3D12, 1:25), Cat# 740267<br>anti-CD4-BV786 (L200, 1:200), Cat# 563914<br>anti-CD8-BUV395 (RPA-T8, 1:400), Cat# 563795<br>anti-CD8-BUV510 (SK1, 1:100), Cat# 563914<br>anti-CD8-APC (SK-1, 1:200), Cat# 345775<br>anti-CD11a-BV510 (HI111, 1:25), Cat# 563480<br>anti-CD28-BV421 (CD28.2, 1:100), Cat# 562613<br>anti-CD38-APC-R700 (HIT2, 1:400), Cat# 564980<br>anti-CD38-BUV737 (HB7, 1:200), Cat# 564686<br>anti-CD39-BV650 (TU66, 33:1), Cat# 563681<br>anti-CD45RA-BUV496 (HI100, 1:800), Cat# 750258<br>anti-CD45RA-BUV737 (HI100, 1:200), Cat# 564442<br>anti-CD69-BUV395 (FN50, 1:50), Cat# 564364<br>anti-CD107a-APC (H4A3, 1:100), Cat# 560664<br>anti-CD127-BUV737 (HIL-7R-M21, 1:50), Cat# 612795<br>anti-CD127-BV421 (HIL-7R-M21, 3:100), Cat# 562436<br>anti-Granzyme B-PE-CF594 (GB11, 1:100), Cat# 562462<br>anti-ICOS-BV711 (DX29, 1:100), Cat# 563833<br>anti-IFN-γ-FITC (25723.11, 1:8), Cat# 340449<br>anti-IL-21-PE (3A3-N2.1, 1:25), Cat# 560463<br>anti-PD-1-PE-Cy7 (EH12.2H7, 1:200), Cat# 561272<br>anti-PD-1-BV786 (EH12.1, 1013122, 3:100), Cat# 563789<br>anti-T-BET-PE-CF594 (O4-46,93533305, 3:100), Cat# 562467<br>anti-TNF-PE-Cy7 (Mab11, 1:400), Cat# 557647<br>ViaProbe (7-AAD, 1:33), Cat# 555816
BioLegend	anti-BCL-2-BV421 (100, 1:200), Cat# 658709<br>anti-CCR7-BV785 (G043H7, 1:50), Cat# 353230<br>anti-CD4-AlexaFluor700 (RPA-T4, 300526, 1:200), Cat# 300526<br>anti-CD25-BV650 (BC96, 1:33), Cat# 302633<br>anti-CD57-BV605 (QA17A04, 1:100), Cat# 563895<br>anti-CD127-BV605 (A019D5, 3:100), Cat# 351334<br>anti-CXCR3-PerCP-Cy5.5 (G025H7, 1:33), Cat# 353714<br>anti-CXCR3-BV510 (G025H7, 3:100), Cat# 353726<br>anti-CXCR5-BV421 (J252D4, 1:100), Cat# 356920<br>anti-IL-2-PerCP-Cy5.5 (MQ1-17H12, 1:100), Cat# 500322<br>anti-Ki67-BV711 (Ki-67, 1:200), Cat# 350516<br>anti-Ki67-PE-Cy7 (Ki67, 1:200), Cat# 350504
Cell Signaling	anti-TCF1-AlexaFluor488 (C63D9, 1:100), Cat# 6444
eBioscience	anti-CD14-APC-eFluor780 (61D3, 1:400), Cat# 47-0149-42<br>anti-CD19-APC-eFluor780 (HIB19, 1:400), Cat# 47-0199<br>anti-CD27-FITC (0323, 1:100), Cat# 11-0279<br>anti-KLRG1-BV711 (13F12F2, 1:50), Cat# 67-9488-42<br>anti-T-BET-PE-Cy7 (4B10, 1:200), Cat# 25-5825<br>anti-TOX-eFluor660 (TRX10, 1:100), Cat# 50-6502<br>anti-EOMES-PerCP-eF710 (WD1928, 1:50), Cat# 46-4877-42<br>Viability Dye (APC-eFluor780 1:200, 1:400) Cat# 65-0865
Invitrogen	anti-CD45RA-PerCP-Cy5.5 (HI100, 3:100), Cat# 45-0458-42
